# Women's recommendations: vacuum extraction or caesarean section for prolonged second stage of labour, a prospective cohort study in Uganda

**DOI:** 10.1111/tmi.13222

**Published:** 2019-03-27

**Authors:** Barbara Nolens, Thomas van den Akker, John Lule, Sulphine Twinomuhangi, Jos van Roosmalen, Josaphat Byamugisha

**Affiliations:** ^1^ Department of Obstetrics and Gynaecology Mulago National Referral Hospital Kampala Uganda; ^2^ Department of Obstetrics and Gynaecology Canisius‐Wilhelmina Hospital Nijmegen The Netherlands; ^3^ Athena Institute Vrije Universiteit Amsterdam The Netherlands; ^4^ Department of Obstetrics and Gynaecology Leiden University Medical Centre Leiden The Netherlands; ^5^ Department of Obstetrics and Gynaecology Kabale University Kabale Uganda; ^6^ School of Medicine College of Health Sciences Makerere University Kampala Uganda

**Keywords:** delivery, vacuum extraction, caesarean section, women's preferences, low‐ and middle‐income countries, accouchement, obstétrique, extraction sous vide, césarienne obstétricale, préférences des femmes, pays à revenu faible ou intermédiaire

## Abstract

**Objectives:**

To investigate what women who have experienced vacuum extraction or second stage caesarean section (CS) would recommend as mode of birth in case of prolonged second stage of labour.

**Methods:**

A prospective cohort study was conducted in a tertiary referral hospital in Uganda. Between November 2014 and July 2015, women with a term singleton in vertex presentation who had undergone vacuum extraction or second stage CS were included. The first day and 6 months after birth women were asked what they would recommend to a friend: vacuum extraction or CS and why. Outcome measures were: proportions of women choosing vacuum extraction *vs*. CS and reasons for choosing this mode of birth.

**Results:**

The first day after birth, 293/318 (92.1%) women who had undergone vacuum extraction and 176/409 (43.0%) women who had undergone CS recommended vacuum extraction. Of women who had given birth by CS in a previous pregnancy and had vacuum extraction this time, 31/32 (96.9%) recommended vacuum extraction. Six months after birth findings were comparable. Less pain, shorter recovery period, avoiding surgery and the presumed relative safety of vacuum extraction to the mother were the main reasons for preferring vacuum extraction. Main reasons to opt for CS were having experienced CS without problems, CS presumed as being safer for the neonate, CS being the only option the woman was aware of, as well as the concern that vacuum extraction would fail.

**Conclusions:**

Most women would recommend vacuum extraction over CS in case of prolonged second stage of labour.

## Abstract (fr)

**Objectifs:**

Investiguer ce que les femmes qui ont subi une extraction sous vide ou une césarienne au second stade du travail recommanderaient comme mode d'accouchement en cas de second stade prolongé du travail.

**Méthodes:**

Une étude de cohorte prospective a été menée dans un hôpital de référence tertiaire en Ouganda. Entre novembre 2014 et juillet 2015, les femmes avec un singleton à terme en présentation de vertex ayant subi une extraction sous vide ou une césarienne de second stade ont été incluses. Le premier jour et six mois après la naissance, on a demandé aux femmes ce qu'elles recommanderaient à une amie: l'extraction sous vide ou la césarienne et pourquoi. Les résultats mesurés étaient: les proportions de femmes choisissant l'extraction sous vide par rapport à la césarienne et les raisons de choisir ce mode d'accouchement.

**Résultats:**

Le premier jour après l'accouchement, 293/318 femmes (92,1%) ayant subi une extraction sous vide et 176/409 (43,0%) femmes ayant subi une césarienne ont recommandé l'extraction sous vide. Parmi les femmes qui avaient accouché par césarienne dans une grossesse précédente et qui avaient eu une extraction sous vide cette fois‐ci, 31/32 (96,9%) ont recommandé l'extraction sous vide. Six mois après la naissance, les résultats étaient comparables. Moins de douleur, une période de convalescence plus courte, l’évitement de la chirurgie et la sécurité relative présumée de l'extraction sous vide pour la mère étaient les principales raisons de préférer l'extraction sous vide. Les principales raisons d'opter pour la césarienne étaient l'expérience d'avoir subi une césarienne sans problèmes, la césarienne étant supposée être plus sûre pour le nouveau‐né, la césarienne étant la seule option dont la femme était au courant, ainsi que la crainte d'un éventuel échec de l'extraction sous vide.

**Conclusions:**

La plupart des femmes recommanderaient l'extraction sous vide plutôt que la césarienne en cas de second stade prolongé du travail.

## Introduction

Prolonged second stage of labour is an important cause of maternal and perinatal morbidity and mortality in low‐ and middle‐income countries (LMIC) 1, 2, 3, 4, 5, 6. Interventions aiming to end prolonged second stage of labour are instrumental vaginal delivery (vacuum extraction and forceps) and caesarean section (CS) 1, 7, 8, 9. Although CS can be a lifesaving procedure and must be available when indicated, the operation may also cause maternal and perinatal morbidity and mortality. Performing CS without strict indication is therefore a major cause of concern 3, 10, 11. Instrumental vaginal delivery (IVD) has many advantages over CS, especially in LMIC, where the risks of surgery are substantial 10, 12. Performing IVD avoids the risks related to anaesthesia and reduces risk of surgery‐related bleeding and infection 13, 14, 15. In addition, delay between decision and birth may be reduced and thereby the risk of uterine rupture or intrauterine foetal death during waiting time 15. Furthermore, the procedure does not result in a uterine scar, with an increased risk of uterine rupture, placenta previa or abnormal invasive placenta in a next pregnancy. This is a particular advantage in settings where many women give birth outside hospital and where these complications are truly life‐threatening 16. The fertility rate in LMIC is often high (5.8 per woman in Uganda during the study period) meaning that, when the first birth is by CS, many ‘trials of labour’ or repeat CS are likely to follow. Other long‐term complications of CS, or complications causing long‐term morbidity, are increased risk of preterm birth in subsequent pregnancies and iatrogenic obstetric fistula 17, 18. Recovery time after IVD is substantially shorter compared to CS and IVD is less costly 10, 19. Therefore, IVD was included as one of the seven signal functions of basic emergency obstetric care and one of the nine signal functions of comprehensive emergency obstetric care (together with CS). Vacuum extraction is recommended as an important management option for prolonged second stage of labour to avoid CS and associated maternal and perinatal morbidity and mortality 15, 20, 21, 22.

Despite its advantages, IVD is hardly used in many LMIC (<1% of institutional births), which is very different from many high‐income European countries that often have frequencies above 15% 23, 24, 25, 26. A cross‐sectional health facility assessment in 40 countries in Latin America, sub‐Sahara Africa and Asia revealed that reasons for not using IVD were equipment related; lack of staff training; issues with authorisation of human resources and the perception amongst staff that no women with an indication for IVD had presented to the health facility 23. Failing to resort to IVD could be a major impediment to the reduction of medically non‐indicated CS and maternal and perinatal morbidity and mortality in LMIC 3, 22. Authorities have declared vacuum extraction the method of choice in modern obstetrics because of its safety for woman and foetus 9, 23. Several projects have been implemented intending to increase the use of vacuum extraction in LMIC, with promising results 27, 28, 29, 30. It is not known, however, whether women find vacuum extraction an acceptable mode of birth, especially in settings where the procedure is uncommon. Studies about women's preferences for mode of birth have only investigated whether women preferred (elective) CS or spontaneous vaginal birth. In those studies, most women preferred vaginal birth above CS 31, 32, 33, 34, 35. The preference of women in case of prolonged second stage of labour has not been studied.

The objective of this study was to investigate what women, who have undergone vacuum extraction or second stage CS, would recommend to their friends in case of prolonged second stage of labour and why.

## Methods

### Study design

A prospective cohort study, consisting of interviews with women who gave birth by vacuum extraction or second stage CS. Interviews were conducted on the first day and 6 months after birth. This study was part of a larger study on clinical and woman‐centred outcomes of vacuum extraction and second stage CS in Mulago Hospital, Uganda. Detailed methods and outcomes were described elsewhere 15, 19.

### Setting

Mulago Hospital is the national referral and main teaching hospital of Uganda, situated in the capital city, Kampala. It is a government hospital with 2700 beds and more than 31 000 births annually. The study was conducted in the main labour ward. Medical care in this ward is free of charge. However, due to shortages women sometimes have to buy medical items outside the hospital (e.g. drugs and urinary catheters). During the study period, the vacuum extraction rate in this ward was 2.6% and the CS rate 31.7%. CS during the second stage of labour in a term singleton pregnancy in vertex presentation occurred to 3.3% of all women. Of women with a term cephalic singleton who had a second stage intervention, 42% had vacuum extraction, 4% had failed vacuum extraction followed by CS and 54% had CS without trial of vacuum extraction 15.

### Participants and period of recruitment

Between 25 November 2014 and 8 July 2015, women with a term, singleton in vertex presentation who had undergone vacuum extraction or CS in the second stage of labour were included, after providing a written informed consent.

### Outcome measures and method of assessment

Outcome measures were: proportions of women recommending vacuum extraction and CS on the first day and 6 months after birth, stratified by mode of birth (vacuum extraction, failed vacuum extraction followed by CS or second stage CS without trial of vacuum extraction). Since unfavourable clinical outcome could influence women's preferences, outcome measures were calculated for all women and also after exclusion of women with unfavourable maternal or perinatal outcome at the moment of interview, defined as: neonate had died before interview, severe maternal complications (re‐laparotomy, hysterectomy and obstetric fistula). Additional outcome measures were reasons for choosing vacuum extraction or CS and frequencies (in percentages), in which those reasons were mentioned, stratified by mode of birth. For reasons of interpretation, clinical information is described when relevant. Method of data collection of clinical outcomes was described elsewhere 15.

On the first day after birth, women were asked what they would recommend to a friend who would need an intervention for prolonged second stage of labour: vacuum extraction or CS (closed question). During a 6 months follow‐up visit or phone call, women were asked what they would recommend to a friend, as well as why they would recommend the chosen mode of birth (open question). Interviews were conducted by trained research assistants who were not performing vacuum extraction or CS themselves. The answers to the open question about why they would recommend the chosen mode of birth were literally recorded into a database by the research assistants. More than one reason per woman was possible. During analysis, reasons given by the women were categorised into ‘main reasons’ (mentioned 15 times or more) and ‘other reasons’ (mentioned less than 15 times). This resulted in five main reasons for choosing vacuum extraction and five main reasons for choosing CS.

### Statistical methods

Baseline characteristics are reported in counts and percentages with *P*‐values comparing vacuum extraction to CS without trial of vacuum extraction. Outcome parameters are reported as counts with percentages. *P*‐values were calculated with two‐sided χ^2^. Data were entered in Microsoft Excel and SPSS version 24 was used for data analysis. *P* < 0.05 was considered statistically significant.

### Study size

A convenience sample was used, since this study was part of a larger study including clinical and woman‐centred outcome after vacuum extraction and second stage CS 15. Sample size for that study was based on expected differences in perinatal death per mode of birth. Missing data per baseline characteristic or outcome parameter varied from 0% to 3.1% and are shown in the tables. Loss to follow‐up is described in Results section.

### Ethical permission

Ethical permission to conduct this study was obtained from the Mulago Hospital Research and Ethics Committee (refnr: MREC 489) and the Uganda National Council for Science and Technology (ref HS1752).

## Results

**Figure 1:**
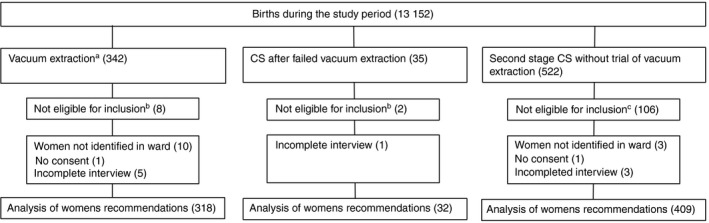
Inclusion process. ^a^One woman had failed vacuum extraction and subsequent forceps delivery (analysed in vacuum extraction group). ^b^One of the following exclusion criteria (more than one could apply): Uterine rupture (2), twin and/or preterm birth (8). ^c^One of the following exclusion criteria (more than one could apply): Maternal death (6), uterine rupture (13) twin, preterm and/or non‐vertex presentation (88).

Of 783 eligible women, 759 (96.9%) participated in the study. Three hundred and eighteen women had vacuum extraction, 32 women had CS after failed vacuum extraction and 409 women had second stage CS without trial of vacuum extraction (Figure 1). One day after birth, 317 (99.7%) women after vacuum extraction, 401 (98.0%) women after second stage CS without trial of vacuum extraction and 32 (100%) women after failed vacuum extraction and subsequent CS had a complete intake interview. Six months after birth, 178 (56.0%) women after vacuum extraction, 226 (55.3%) women after CS without trial of vacuum extraction and 22 (68.8%) women after failed vacuum extraction and subsequent CS could be interviewed.

Table 1 shows socio‐demographic characteristics. Ninety‐nine of four hundred and nine (24.2%) women who had CS without trial of vacuum extraction had a previous CS *vs*. 32/318 (10.1%) women who had vacuum extraction (*P* < 0.001). Other characteristics were not statistically different between the groups.

**Table 1:** Characteristics of participants

Mode of birth	Vacuum extraction (318)		CS without trial of vacuum extraction (409)		CS after failed vacuum extraction (32)	
	*n*	%	*n*	%	*n*	%
Parity						
Nulliparous	175	55.0	207	50.6	22	68.8
Parous	137	43.1	202	49.4	10	31.3
Missing data	6	1.9	0	0	0	0
Previous CS						
Yes	32	10.1	99	24.2	5	15.6
No	279	87.7	310	75.8	27	84.4
Missing data	7	2.2	0	0	0	0
Education						
None	3	0.9	9	2.2	1	3.1
1–6 years	76	23.9	91	22.2	5	15.6
7–12 years	209	65.7	271	66.3	22	68.8
>12 years	25	7.9	32	7.8	4	12.5
Missing data	5	1.6	6	1.5	0	0
Occupation						
Employed	117	36.8	170	41.6	15	46.9
Student	3	0.9	5	1.2	0	0
Unemployed	191	60.1	228	55.7	16	50.0
Missing data	7	2.2	6	1.5	1	3.1
Age						
Mean age	23.3	SD 5.2	23.9	SD 5.3	23.4	SD 5.3
<20 years	78	24.5	88	21.5	5	15.6
≥20 years	235	73.9	320	78.2	27	84.4
Missing data	5	1.6	1	0.2	0	0

CS, Caesarean section.

During the interview on the first day after birth, the majority of women who had vacuum extraction (293/318; 92.1%) would recommend this procedure. Almost half of women who had CS (176/409; 43.0%) would recommend vacuum extraction rather than CS (Table 2). When women with unfavourable outcome were excluded, these figures did not change (Table S1). Of 32 women who had experienced CS in a previous pregnancy and vacuum extraction during this study, 31 women (96.9%) would recommend vacuum extraction to a friend rather than CS. During the follow‐up interview at 6 months after birth, the answers were similar to those on the first day after birth (Table 2).

**Table 2:** Women's recommendations in case of second stage intervention

Mode of birth	Vacuum extraction		CS without trial of vacuum extraction		CS after failed vacuum extraction	
	*n* (318)	%	*n* (409)	%	*n* (32)	%
Recommendation on first day after birth						
Vacuum extraction	293	92.1	176	43.0	14	43.8
Caesarean section	24	7.5	225	55.0	18	56.3
Missing data	1	0.3	8	2.0	0	0
Recommendation at 6 months after birth	*n* (178)	%	*n* (226)	%	*n* (22)	%
Vacuum extraction	160	89.9	100	44.2	9	40.9
Caesarean section	14	7.9	123	54.4	13	59.1
No preference	4	2.2	3	1.3	0	0

CS, caesarean section.

### Main reasons for recommending vacuum extraction

Reasons why women would recommend vacuum extraction are shown in Table 3. Less pain was the most important reason for recommending vacuum extraction, especially in women who had experienced CS and would recommend vacuum extraction. A short recovery period, avoiding surgery, the presumption that vacuum extraction is safer for the mother and having experienced vacuum extraction without problems were other frequently mentioned reasons.

**Table 3:** Reasons for recommending vacuum extraction or CS at 6 months after birth

Mode of birth	Vacuum extraction (178)		CS without trial of vacuum extraction (226)		CS after failed vacuum extraction (22)		All women (426)	
Women who recommended vacuum extraction	*n* (160)	%*	*n* (100)	%*	*n* (9)	%*	*n* (269)	%*
**Reasons for recommending vacuum extraction**								
Less pain during/after vacuum extraction	50	31.3	54	54.0	6	66.7	110	40.9
Short recovery, no limitations	28	17.5	14	14.0	3	33.3	45	16.7
Vacuum extraction is like normal delivery/no operation or scar	27	16.9	13	13.0	0	0.0	40	14.9
Vacuum extraction is safer for mother	20	12.5	17	17.0	0	0.0	37	13.8
I had no problems with vacuum extraction	28	17.5	0	0.0	0	0.0	28	10.4
Other reason†	28	17.5	8	8.0	0	0.0	36	13.4
Women who recommended CS	*n* (14)	%*	*n* (123)	%*	*n* (13)	%*	*n* (150)	%*
**Reasons for recommending CS**								
I had no problems with CS	0	0.0	44	35.8	2	15.4	46	30.7
CS is safer for baby	8	57.1	30	24.4	2	15.4	40	26.7
CS is the only option I know	0	0.0	21	17.1	0	0.0	21	14.0
Vacuum extraction may fail	0	0.0	12	9.8	9	69.2	21	14.0
CS is safer for mother	2	14.3	18	14.6	0	0.0	20	13.3
Other reason†	9	64.3	20	16.3	2	15.4	31	20.7
Women who did not make a choice	4/178	2.2‡	3/226	1.3‡	0/22	0.0‡	7/426	1.6‡

CS, caesarean section.

* Women who gave this reason as percentage of women who recommended this mode of birth per mode of birth group (more than one reason per woman possible).

† Other reasons for recommending vacuum extraction: vacuum extraction is easier/less complicated (12); CS is scary (10); vacuum extraction saves lives (5); vacuum delivery is faster (4); vacuum extraction is safer for baby (3); I've heard bad stories about CS (1); concern about sexual activity after CS (1). Other reasons for recommending CS: CS saves lives (11); vacuum extraction is scary (8); CS is faster (5); less pain during/after CS (3); good care after CS (2); the ones helping you have no experience in vacuum extraction (1).

‡ Percentage of women who did not make a choice per mode of birth group.

Quotes that illustrate reasons for recommending vacuum extraction are shown below:

“I would advise vacuum extraction to a friend, because I have experienced both and CS was too painful compared to vacuum. I had CS on my first born and it was terrible. But now (after vacuum extraction) I am very OK.”

*23‐year‐old housewife, now P2, gave birth to 3.1 kg girl by vacuum extraction*.

“After vacuum extraction you can work. After CS it may take six months.”

*19‐year‐old business woman, now P1, gave birth to 3.1 kg boy by vacuum extraction*.

“I would recommend vacuum extraction because I recovered so fast compared to my friends who were cut.”

*20‐year‐old business woman, now P1, gave birth to 3.1 kg girl by vacuum extraction*.

“Vacuum extraction seems normal, while with CS one is cut open.”

*17‐year‐old bar attendant, now P1, gave birth to 2.5 kg girl by vacuum extraction*.

“Vacuum extraction prevents operation and is not so painful.”

*30‐year‐old restaurant attendant, now P3, gave birth to 3.7 kg girl by CS*.

“One does not have to go through the trauma of (operating) theatre.”

*22‐year‐old housewife, now P1, gave birth to 3.5 kg girl by vacuum extraction*.

“CS is total deformity.”

*19‐year‐old hairdresser, now P1, gave birth to 3.0 kg boy by CS*.

“Vacuum extraction saved me and my baby. Some people die during CS.”

*30‐year‐old housewife, now P4, gave birth to 4.0 kg boy by vacuum extraction*.

### Main reasons for recommending CS

The most frequently mentioned reasons for choosing CS were: having experienced CS without problems; CS presumed as being safer for the neonate; CS being the only option the woman was aware of, concern that vacuum extraction may fail and CS presumed as being safer for the mother:

“I would recommend CS, because I don't know vacuum extraction.”

*20‐year‐old hairdresser, now P1, gave birth to 2.9 kg girl by CS*.

“I don't know vacuum extraction; the baby might get damage to the head.”

*Housewife, now P1, gave birth to 3.7 kg boy by CS*.

“Vacuum extraction may fail and when they take you to (operating) theatre it's too late.”

*18‐year‐old business woman, now P1, gave birth to 3.1 kg boy by CS*.

“I had failed vacuum and it was very painful.”

*20‐year‐old hairdresser, now P2, gave birth to 3.6 kg boy by CS after failed trial of vacuum extraction. Neonate was in neonatology unit for 11 days for suspected birth asphyxia, but showed normal development at 6 months after birth*.

“CS can save baby and mother. In the process of vacuum extraction, one can die, mother or baby.”

*34‐year‐old hairdresser, now P3, gave birth to 4.2 kg girl by CS*.

### Other reasons for recommending vacuum extraction or CS

Some women recommended vacuum extraction but were concerned about trauma to the neonate as well. Other women were rather concerned about perinatal outcome after CS:

“I would recommend vacuum extraction, but only if there is an assurance that the baby's brain will not be damaged.”

*20‐year‐old trader, now P2, had one previous CS and gave birth to 3.0 kg boy by vacuum extraction. Neonate had no signs of brain damage at birth (Apgar score 8‐9) or at 6 months follow up*.

“Maybe vacuum extraction saves babies’ lives, since it is faster.”

*34‐year‐old business woman, now P4, had one previous CS. Gave birth to a stillborn 3.0 kg boy by (repeat) CS. Intrauterine foetal death occurred during waiting time for CS*.

“When babies are born vaginally, they breathe better.”

*30‐year‐old housewife, now P5, gave birth to 4.0 kg boy by CS*.

Vacuum extraction perceived as being scary was mentioned by eight women:

“I witnessed vacuum extraction and it was horrible.”

*19‐year‐old housewife, now P1, gave birth to 2.7 kg boy by CS*.

One woman mentioned:

“The ones helping you have no experience in vacuum extraction.”

*35‐year‐old housewife, now P2, had one previous CS, gave birth to 2.8 kg boy by (repeat)CS*.

The (higher) costs of CS were mentioned by one woman:

“If financially stable they can do CS, but if not, they should do vacuum.”

*26‐year‐old housewife, now P3, gave birth to 3.2 kg girl by vacuum extraction*.

Six months after birth, only eight out of 161 (5.0%) women who had given birth by vacuum extraction with good outcome (neonate alive and no severe maternal complications) recommended CS, while 78 out of 193 (40.4%) women who had undergone CS with good outcome would recommend vacuum extraction to a friend. Reasons for recommending CS after having experienced vacuum extraction with good outcome were (with number of women who mentioned this reason in brackets) pain during vacuum extraction (2); ‘My baby had to go to neonatology unit’ (1) (The neonate was in the neonatology unit for suspected birth asphyxia and showed normal development at 6 months after birth.); ‘It felt bad to see my baby's head swollen’ (1) (Subgaleal haemorrhage was suspected. The neonate had phototherapy and showed normal development at 6 months after birth.); Vacuum extraction was scary (2); Complications after vacuum extraction (1) (Mother and neonate went home after 1 day, no complications noted at discharge and at 6 months follow‐up.)

## Discussion

The vast majority of women who had experienced vacuum extraction would recommend this mode of birth above CS in case of prolonged labour. Nearly half of the women who experienced CS would also recommend vacuum extraction. Main reasons for choosing vacuum extraction were experiencing less pain, having a shorter recovery period, avoiding surgery and vacuum extraction being presumed as being safer for the mother. Main reasons for recommending CS were having experienced CS without problems, CS presumed as being safer for the neonate, CS being the only option the woman was aware of and concern that vacuum extraction may fail.

These results show that most women perceive vacuum extraction as an acceptable intervention for prolonged second stage of labour. In case they had experienced the procedure, they clearly preferred this intervention above CS. These results are in line with previous findings from the same setting: 91% of the women after vacuum extraction were satisfied about their birthing experience 19. A study from Argentina found that only 6% of the healthy pregnant nulliparous women (without indication for CS) in the public sector preferred CS above vaginal birth 33. In a study from Italy, 94% of the parous women without previous CS would prefer to have a vaginal birth in a next pregnancy compared to 60% of the parous women with a previous CS 34. Reasons for preferring vaginal birth in that study were not wanting to be separated from the neonate, shorter hospital stay and faster postpartum recovery.

Reasons for choosing vacuum extraction in our study are supported by results of studies in the same setting 15, 19: after vacuum extraction, compared to after CS, pain scores were lower up to 6 weeks after birth and more women were able to work at 6 weeks after birth 19. Vacuum extraction was indeed safer for the mother 15: risk of severe maternal complications (maternal death, uterine rupture while waiting for procedure, hysterectomy and re‐laparotomy) was 0.8% (3/358) in women who had had (trial of) vacuum extraction as compared to 4.2% (18/425) in women who had undergone second stage CS. During or after CS 5/425 (1.2%) of women died, none (0/358) after (trial of) vacuum extraction 15.

‘Vacuum extraction is like normal birth’ or ‘I do not want an operation or scar’ were important reasons to choose vacuum extraction. This might be of particular importance to women in countries where having had CS is seen as abnormal, ‘a significant subtraction from womanhood’ or even as ‘the devil's work’ or ‘a sign of marital infidelity’ 31, 36.

An important reason for recommending CS is the belief that CS is safer for the neonate. However, this is not supported by publications from Uganda and the United States 15, 37. In our setting (Uganda), a study of clinical outcome of 757 neonates after either second stage CS or (trial of) vacuum extraction showed that perinatal outcome and outcome at 6 months after birth was comparable. Occurrence of perinatal death was 45/410 (11.0%) in the CS group and 29/347 (8.4%) in the vacuum extraction group (*P* = 0.227). Occurrence of intra uterine foetal death during waiting time for CS was 18/410 (4.4%) and for vacuum extraction 3/347 (0.9%, *P* = 0.003) 15.

It is clear that many women are not aware of the risks and benefits of vacuum extraction *vs*. CS. This is an important knowledge gap for pregnant women and possibly for health care providers in this setting. In the situation of prolonged second stage with a clear indication for a vacuum extraction, this option should be promoted as the option of first choice. Women will have to be explained risks and benefits of vacuum extraction, also in relation to CS, and should be asked to provide consent.

Other reported reasons for choosing CS, such as having experienced CS without problems and CS being the only option a woman was aware of, would probably be mentioned less often if women had been better informed.

The reason ‘Vacuum extraction may fail’ is indeed a realistic concern. In this cohort the failure rate was 9.1% (32/350, Figure 1), comparable to failure rates elsewhere 9. Interestingly, 14/32 (43.8%) of the women after failed vacuum extraction would still recommend vacuum extraction. Training and adhering to clinical guidelines are important in keeping failure rates as low as possible.

Although most women in our study would recommend vacuum extraction above CS, vacuum extraction is not always a realistic management option. In some areas, neither CS nor vacuum extraction is available, while in other areas vacuum extraction is not available and CS rates are alarmingly high 11, 23, 25. Such situations clearly represent a missed opportunity. Inexperience or inadequate skills in performing vacuum extraction have been associated with greater frequency of CS use 22. Implementation programs aiming at increasing the use of vacuum extraction by training of staff, supply of equipment, development of guidelines, audit of indications for CS and vacuum extraction have shown to be effective 21, 27, 28, 29, 30. More such programs are needed to ensure that women who have an indication for vacuum extraction benefit from the procedure.

### Strengths and limitations

A strength of this study is that it addresses an important knowledge gap. Nearly all eligible women accepted to be included, minimising selection bias. An additional strength is that not only women who had experienced vacuum extraction, but also women who had undergone second stage CS or who had had a failed trial of vacuum extraction were interviewed. Some women in this study may have felt that they should give a response in favour of the care option they received, although 44% of the women after CS recommended vacuum extraction. Only interviewing women after vacuum extraction would give results that would be difficult to interpret. Eventual bias is expected to be in the same direction for the different groups and is not expected to change the conclusions of the study. The observational design comes with obvious limitations. The baseline characteristic ‘previous CS’ was more frequent in women who had given birth by CS, and this might have introduced bias. Losses to follow‐up at 6 months are a limitation and could have introduced additional bias, although losses to follow‐up were comparable between the different groups. Although participants were from different socioeconomic backgrounds and educational levels, the study was performed in a single health facility in an urban setting. Findings may be generalisable to similar settings, but repetition of our study in other similar and different settings must be encouraged.

In conclusion, the majority of women in this tertiary referral centre in Uganda, would recommend vacuum extraction over CS in case of prolonged second stage of labour.

These findings are in line with literature that vacuum extraction should be the procedure of choice in prolonged second stage of labour to avoid CS, unless a clear contraindication is present. Implementation programs are much needed to make vacuum extraction a realistic management option for all women requiring this procedure.

## Declarations

BN serves on an advisory committee for the development of a new vacuum extractor product by Clinical Innovations (South Murray, UT, USA). This company had no role in either the present study or the content of the manuscript. The authors have no other conflict of interest.

## Supporting information


**Table S1.** Women's recommendations in case of second stage intervention. Selection: good maternal and perinatal outcome
