# Supplementary material for: Women's recommendations: vacuum extraction or caesarean section for prolonged second stage of labour, a prospective cohort study in Uganda
Source: Trop Med Int Health. 2019 Mar 27;24(5):553–62. doi: 10.1111/tmi.13222 (PMC6850599; doi:10.1111/tmi.13222)
Supplement: Supplementary file 1 — Table S1. Women's recommendations in case of second stage intervention. Selection: good maternal and perinatal outcome [file TMI-24-553-s001.docx]

| **Supplementary table** Women’s recommendations in case of second stage intervention. Selection: good maternal and perinatal outcome^a^ | | | | | | |
| --- | --- | --- | --- | --- | --- | --- |
| **Mode of birth** | **Vacuum extraction**  **(289)** | | **CS without trial of vacuum extraction**  **(362)** | | **CS after failed vacuum extraction**  **(28)** | |
| **Recommendation on first day after birth^b^** | **n=289** | **%** | **n=362** | **%** | **n=28** | **%** |
| Vacuum extraction | 271 | 93.8 | 154 | 42.5 | 13 | 46.4 |
| Caesarean section | 17 | 5.9 | 201 | 55.5 | 15 | 53.6 |
| Missing data | 1 | 0.3 | 7 | 1.9 | 0 | 0.0 |
| **Recommendation at six months after birth^c^** | **n=161** |  | **n=193** |  | **n=17** |  |
| Vacuum extraction | 151 | 93.8 | 78 | 40.4 | 8 | 47.1 |
| Caesarean section | 8 | 5.0 | 112 | 58.0 | 9 | 52.9 |
| No preference | 2 | 1.2 | 3 | 1.6 | 0 | 0.0 |
| a: defined as neonate had died before interview, severe maternal complications (re-laparotomy, hysterectomy, obstetric fistula)  When outcome in table 2 is compared to outcome in this table, using p-values, all p-values are ≥ 0.05 | | | | | | |
